# Polymyxin B containing polyion complex (PIC) nanoparticles: Improving the antimicrobial activity by tailoring the degree of polymerisation of the inert component

**DOI:** 10.1038/s41598-017-09667-3

**Published:** 2017-08-24

**Authors:** Ignacio Insua, Laimdota Zizmare, Anna F. A. Peacock, Anne Marie Krachler, Francisco Fernandez-Trillo

**Affiliations:** 1 School of Chemistry, University of Birmingham, B15 2TT Birmingham, UK; 2 School of Biosciences, University of Birmingham, B15 2TT Birmingham, UK; 3 Institute of Microbiology and Infection, University of Birmingham, B15 2TT Birmingham, UK; 4 Department of Microbiology and Molecular Genetics, University of Texas McGovern Medical School at Houston, Houston, TX 77030 USA

## Abstract

Here, we describe the preparation and characterisation of polyion complex (PIC) nanoparticles containing last resort antimicrobial polymyxin B (Pol-B). PIC nanoparticles were prepared with poly(styrene sulphonate) (PSS) as an inert component, across a range of degrees of polymerisation to evaluate the effect that multivalency of this electrolyte has on the stability and antimicrobial activity of these nanoparticles. Our results demonstrate that while nanoparticles prepared with longer polyelectrolytes are more stable under simulated physiological conditions, those prepared with shorter polyelectrolytes have a higher antimicrobial activity. Tailoring the degree of polymerisation and the ratio of the components we have been able to identify a formulation that shows a sustained inhibitory effect on the growth of *P*. *aeruginosa* and can reduce the number of viable colonies of this pathogen over 10,000 times more effectively than our previously reported formulation.

## Introduction

Polyion complex (PIC) particles, also known as polyelectrolyte complexes (PECs) or interpolyelectrolyte complexes (IPECs), are soft colloids formed by the spontaneous aggregation of oppositely charged polyelectrolytes in aqueous media^1–3^. Due to their straightforward preparation and versatility, PIC particles are particularly suited for the delivery of charged (bio)molecules (e.g. nucleic acids and proteins), which are prevalent in nature^3–5^. This way, delivery vehicles can be formulated without the need to introduce chemical modifications to these (bio)molecules and as a result, the biological activity of these therapeutics should be maintained upon release. PIC particle formation and stability are dominated by entropic effects^6^ making them ideal for the delivery of “large” and densely charged macromolecules such as nucleic acids^3, 7, 8^. More challenging is the use of PIC particles for the delivery of proteins^9, 10^, peptides^11^ or small molecules^3^, which often lack the required charge density to form stable PIC particles. However, many of the characteristics of PIC particles makes them ideally suited for the delivery of antimicrobial peptides, which are small cationic peptides that have been harnessed as last-resort antimicrobials or as alternatives to current antibiotics^12–14^. The cationic residues in antimicrobial peptides can be shielded within PIC particles^15–18^, thus minimising the toxicity towards the host associated with the cationic and amphiphilic nature of antimicrobial peptides^12–14, 19, 20^. With the increasing concern of antimicrobial resistance, new ways of employing antimicrobials are highly desired, either through repurposing “old” antibiotics, or finding better ways of using current antimicrobials^21, 22^. In this regard, encapsulation of antimicrobials in polymeric nanomaterials can offer several advantages, including decreasing toxicity, improving bioavailability, increasing circulation time, or improving penetration in infected tissues^23–25^. In applications such as wound healing, a sustained release of the antimicrobial may be desirable, to maintain a therapeutic level of the antimicrobial throughout the treatment.

In our laboratories, we have recently developed PIC nanoparticles loaded with the clinically approved antibiotic polymyxin B (Pol-B)^26–30^ by complexation with FDA approved poly(styrene sulphonate) (PSS)^15^. Both Pol-B and PSS are strong polyelectrolytes (*pK*_*a*_ *H* 9–10^31^ and *pK*_*a*_ ∼ −2.5^32^ respectively) and fully charged at neutral pH, making them ideal components for the formulation of PIC nanoparticles. These Pol-B containing nanoparticles displayed a moderate antimicrobial effect against *Pseudomonas aeruginosa*, an opportunistic Gram-negative bacterium, for which Pol-B is currently used as a last resort antimicrobial. Based on the correlation between the stability of PIC particles and the net charge of their consisting polyelectrolytes, the formulation of Pol-B with shorter polyelectrolytes can potentially result in more permeable PIC nanoparticles with stronger antimicrobial properties.

In this communication, we describe how the antimicrobial activity of these Pol-B containing PIC nanoparticles has been significantly improved by tailoring the molecular weight of the inert component, PSS. First, we investigate the effect that reducing the degree of polymerisation of PSS (i.e. molecular weight) has on the stability of the formed nanoparticles under simulated physiological conditions and in growth medium. Then, we evaluate the antimicrobial activity against *P*. *aeruginosa* of the resulting Pol-B containing PIC nanoparticles. Our results indicate that nanoparticles prepared with shorter PSS are less stable under simulated physiological conditions but result in significantly higher antimicrobial activities, and leads to the identification of a formulation that shows a sustained inhibitory effect on the growth of *P*. *aeruginosa* and that reduces the number of viable colonies of this pathogen more effectively than our previously reported formulation.

## Experimental

### Materials

Polymyxin B sulphate (Pol-B) was purchased from Alfa Aesar®. Sodium poly(styrene sulphonate) (PSS) of 32, 17 and 10 kDa average Mw (GPC standards), 4-(2-hydroxyethyl)piperazine-1-ethanesulfonic acid (HEPES), phosphotungstic acid hydrate, agar and Luria Bertani (LB) broth (Miller) were bought from Sigma-Aldrich Ltd. Nylon 0.45 µm syringe filters were purchased from Camlab Ltd. Carbon-coated nickel TEM grids (200 mesh) were purchased from Agar Scientific Ltd. Dulbecco’s phosphate buffered saline (DPBS), 9.5 mM in phosphate and without Ca^2+^ and Mg^2+^, was bought from Lonza.

### Instrumentation

Dynamic light scattering (DLS) and ζ-potential measurements were carried out in a Zetasizer Nano ZSP (Malvern Instruments Ltd.) stabilised at 37 °C. DLS was read at 173° (backscattering) for 60 seconds in triplicate and ζ-potentials were recorded 30 times at 140 V. Transmission Electron Microscopy (TEM) images of PIC nanoparticles were acquired on a JEM-1200EX (JEOL USA Inc.). PIC nanoparticle size was measured from TEM micrographs using ImageJ software (1.48 v) and measuring each nanoparticle twice: both in their longest and shortest diameters. A Loctite® LED flood array (Henkel Ltd.) operating at 405 nm was used to sterilise the samples studied in the bacterial growth experiment. A FLUOstar Omega (BMG Labtech GmbH.) microplate reader was used to incubate and measure the optical density at 600 nm (OD_600_) in the bacterial growth experiments. Pictures of agar plates were taken on a ChemiDoc^TM^ MP imaging system (Bio-Rad laboratories Inc.).

### Preparation of PIC nanoparticles

PIC nanoparticles from Pol-B and PSS were prepared following the protocol previously described by our group^15, 33^. The different formulations of PIC nanoparticles are defined by their [n+/n−] ratio, which represents the proportion of ammonium groups in Pol-B over acidic groups in PSS. As a representative example, for the preparation of PIC nanoparticles at 0.5 [n+/n−] ratio, solutions of Pol-B (0.25 mM) and PSS (2.5 mM in monomer units) were prepared separately in 5 mM HEPES buffer at pH 7.4. Then, both stock solutions were filtered through 0.45 µm nylon syringe filters and mixed in equal amounts drop-wise under stirring. PIC nanoparticles at other [n+/n−] ratios were obtained by changing the concentration of the Pol-B stock solution and mixing with 2.5 mM PSS following the same protocol. After 24 h, the polyion mixtures were characterised by DLS and ζ-potential without prior dilution nor filtration (Table S1†).

### TEM imaging of PIC nanoparticles

For TEM analysis, 5 µL of a suspension of PIC nanoparticles were deposited on the surface of the TEM grid and it was left to dry at room temperature covered from dust. Once dried, 5 µL of a 1 mg·mL^−1^ solution of phosphotungstic acid in water were deposited on the grid to stain the nanoparticles for a minute, after which the excess staining solution was removed. Finally, the grid was dried as before.

### Stability of PIC nanoparticles in simulated physiological conditions

To a sample of PIC nanoparticles (1 mL), prepared as described above, 182 μL of a 1 M solution of NaCl in water was added and the mixture was incubated at 37 °C to obtain physiological osmotic pressure and temperature. Every hour, the sample was analysed by DLS as described above.

### Pol-B diffusion during dialysis

2.5 mL (or 1.2 mL for Amicon Ultra centrifugal filter units) of a 175 μM solutions of Pol-B in 5 mM HEPES buffer pH 7.4 were transferred into pre-conditioned dialysis devices (Float-a-Lyzer® G2 or Amicon), and were dialysed against 15 mL (or 8 mL for Amicon) of DPBS at 37 °C under stirring. 500 μL of dialysate were taken at different time points and replaced with fresh DPBS. The dialysate samples were analysed by HPLC using an adaptation of the method previously reported by Orwa *et al*.^34^, using a 50:23:22:5 mixture of 50 mM Na_2_SO_4_ in H_2_O: H_2_O: AcCN: 5% (v/v) dilution of 85% (m/m) H_3_PO_4_ in H_2_O running at 1 mL/min. 3 replicates were studied under the same conditions. At every time point, the area under the peak of Pol-B found in each dialysate sample was normalised to the area found in a non-dialysed solution of Pol-B prepared at the same concentration and diluted with 15 mL of DPBS (100% release).

### Antimicrobial evaluation of PIC nanoparticles: Growth curves

100 μL aliquots of *P*. *aeruginosa* PAO1V^35^ cultures in LB broth (OD_600_ = 0.2) were loaded in the designated positions of a 96-well microplate. These bacterial cultures were mixed with 100 μL of PIC nanoparticles or free Pol-B in 5 mM HEPES buffer pH 7.4 to compare the antimicrobial activity of free and complexed (within PIC nanoparticles) Pol-B at the same concentration. Alternatively, these bacterial cultures were mixed with 100 μL of 5 mM HEPES buffer pH 7.4 as positive growth control or 1.25 mM (in monomer units) PSS in 5 mM HEPES buffer pH 7.4. to evaluate the effect of the polymers alone. Finally, 200 μL of a 1:1 mixture of the LB broth and 5 mM HEPES buffer pH 7.4 were also loaded in the microplate to confirm the sterility of the medium. All samples were prepared in triplicate, with mean values and standard deviations reported. The microplate was incubated at 37 °C and orbital shaking, and the OD_600_ was monitored every 30 min for 24 h. The samples of PIC nanoparticles, free Pol-B, PSS and 5 mM HEPES buffer 7.4 were sterilised prior to this experiment by exposure to blue light (405 nm) for 2 h at a distance of 15 cm from the light source.

### Antimicrobial evaluation of PIC nanoparticles: CFU counting

After 24 h, 10 µL of the samples from the ‘growth curve’ experiment (see above) were spotted in triplicate on LB agar plates in serial dilution, from undiluted to 10^2^, 10^4^ and 10^6^-fold dilutions. These agar plates were then incubated at 37 °C. After 18 h of incubation, the agar plates were photographed and bacterial concentrations in CFU·mL^−1^ were calculated from the highest dilution that showed individual colonies.

## Results and Discussion

**Figure 1:**
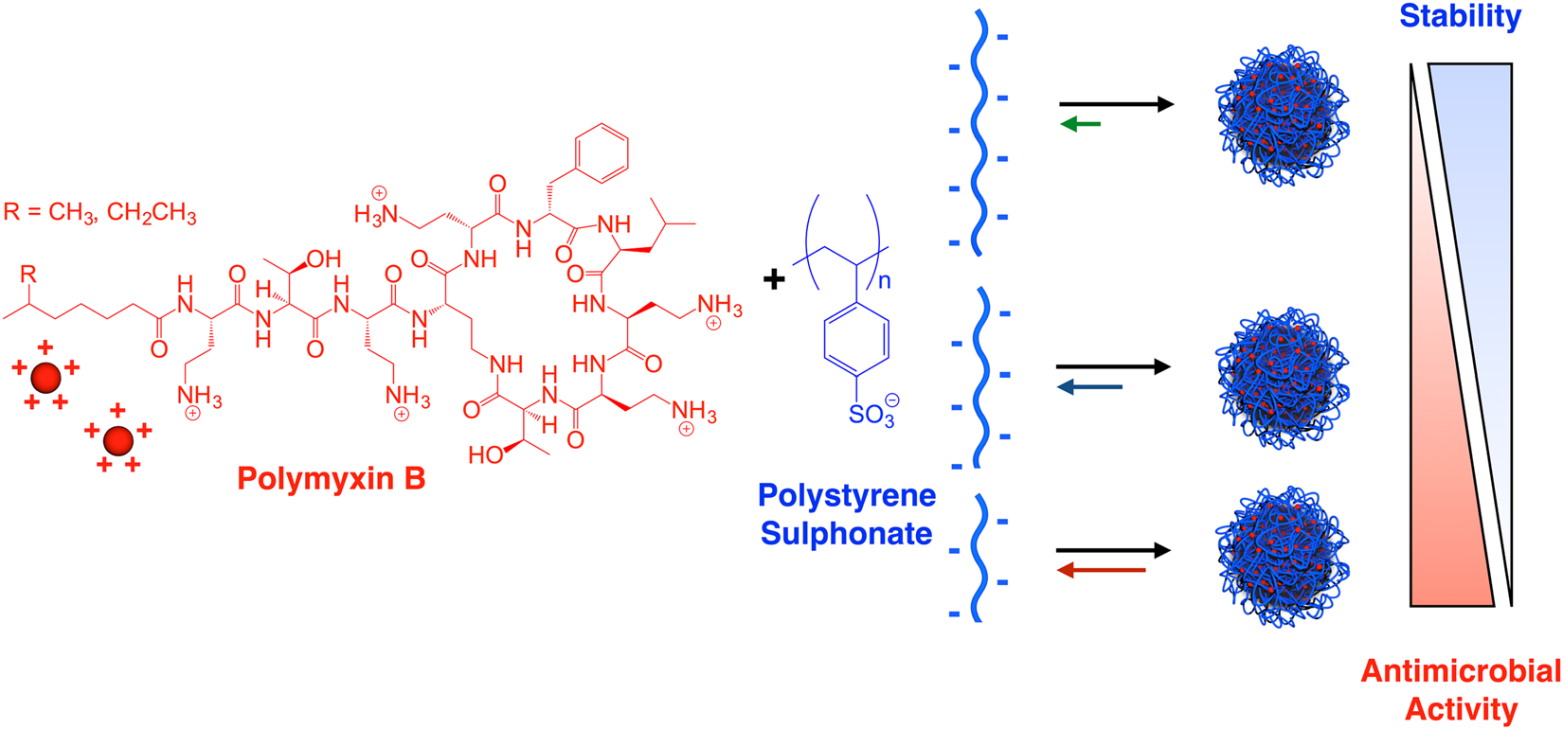
Schematic representation of the formation of PIC nanoparticles by self-assembly of cationic polymyxin B with poly(styrene sulphonate) (PSS) and effect of the change in multivalency.

We have recently reported the preparation of PIC nanoparticles via self-assembly of the cationic antibiotic polymyxin B (Pol-B) and the polyanion poly(styrene sulphonate) (PSS)^15^. These nanoparticles displayed a formulation-dependent antimicrobial effect against *P*. *aeruginosa*, and can be an alternative delivery vehicle to the conventional use of antibiotics. Although the complexation of Pol-B is challenging due to its low net charge or multivalency (Fig. 1)^36^, this antibiotic was successfully assembled into PIC nanoparticles using a very high molecular weight PSS (70 kDa). The low multivalency of pentacationic Pol-B was compensated with PSS chains bearing *ca*. 340 charges on average, forming a highly multivalent network of electrostatic cross-links that stabilised these PIC nanoparticles. However, these nanoparticles showed moderate antimicrobial activity and our results suggested that the antimicrobial activity of the nanoparticles was related to the stability of the nanoparticles in the assay growth medium.

Since the stability of PIC particles in solution strongly depends on the multivalency of their constitutive polyelectrolytes^3^, we propose here that the complexation of Pol-B with shorter PSS would lead to less stable polyelectrolyte complexes with potentially faster Pol-B release profiles and higher antimicrobial activities. To test this hypothesis, we investigated the assembly of PIC nanoparticles from Pol-B and PSS with average molecular weights below 70 kDa. We selected commercial PSS sources of 30 and 15 kDa to test polymers with approximately half and a fourth of the Mw previously reported. Moreover, the shortest commercially available PSS (10 kDa) was also tested (Table 1).

**Table 1:** Polyanions investigated in this work.

Code^a^	Mp^b^	Mw^b^	DP^c^	*Đ* ^b^
PSS_H_	29,500	29,100	141	<1.2
PSS_M_	15,800	14,900	72	<1.2
PSS_L_	10,200	10,600	51	<1.2

^a^PSS subindexes stand for high (H), medium (M) and low (L) DP. ^b^Data provided by the manufacturer. ^c^Degree of polymerization based on the Mw.

**Figure 2:**
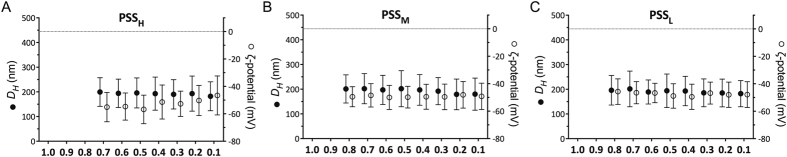
Hydrodynamic diameter (*D*_*H*_, ⦁) and ζ-potential (○) of PIC nanoparticles prepared at different [n+/n−] ratios from Pol-B and PSS_H_ (**A**), PSS_M_ (**B**), and PSS_L_ (**C**). Blank spaces indicate unstable formulations that flocculated. Each value represents the mean diameter or charge of the only size or charge population fitted by the software ± its standard deviation.

Pol-B was therefore mixed with each PSS in ten different proportions (i.e. charge ratios), denoted as [n+/n−], indicating the number of ammonium groups in Pol-B divided by the number of sulphonates in PSS present in each nanoparticle formulation. The evaluation of a range of charge ratios is critical because this parameter not only dictates the dose of antibiotic loaded inside these nanoparticles, but it can also affect their stability in solution^3^. Nanoparticles were used as prepared without further purification, in a similar fashion to those previously reported using 70 kDa PSS^15^. In the absence of additional steps to stabilise the particles (e.g. cross-linking), PIC particles are always present as a mixture with the individual components. Any attempt to remove loosely bound drug will result in the destabilisation of the system, particularly for PIC particles like the ones reported here that employ polyelectrolytes with low multivalency. We anticipate that, upon application, any Pol-B that is free or loosely bound to the particles would have an immediate effect on *P*. *aeruginosa*, while the nanoparticles would act as a reservoir of the drug, maintaining therapeutic levels of the antimicrobial. The range of [n+/n−] ratios explored here was selected from our previous observations with a 70 kDa PSS and Pol-B, which formed unstable complexes that flocculated at values of [n+/n−] ≥ 1 due to the complete neutralisation of the PSS chains by Pol-B^15^. Thus, we decided to explore [n+/n−] ratios between 1.0 and 0.1 and the resulting nanoparticle formulations were characterised by DLS and ζ-potential (Fig. 2, Table S1†). Colloidal particles were obtained for most of these formulations even when as few as 0.1 equivalents of Pol-B were employed. As observed for other polyelectrolytes^37, 38^, we were not able to form PIC nanoparticles with any of the PSS tested at [n+/n−] ratios around equimolar range (i.e. 1.0, 0.9, and even 0.8 for the highest Mw PSS tested here, PSS_H_), which led to unstable aggregates that flocculated due to charge neutralisation (Fig. 2).

**Figure 3:**
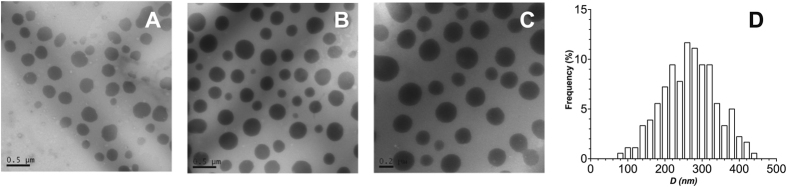
TEM micrographs of PIC nanoparticles prepared from Pol-B and PSS_H_ at a 0.4 [n+/n−] ratio (**A**–**C**) and the size distribution of these nanoparticles found by TEM (**D**). Scale bars from left to right: 0.5 (**A**), 0.5 (**B**) and 0.2 μm (**C**).

All nanoparticles prepared from PSS showed almost identical hydrodynamic diameters (*D*_*H*_) and ζ-potentials regardless of the DP of the polymer, with average values of 192 nm (±4%)^39^ in *D*_*H*_ and −49.6 mV (±6%)^39^ in charge (Fig. 2). These values were in agreement with those previously reported for 70 kDa PSS^15^. Representative formulations were analysed using TEM to assess whether such loose complexes would still retain morphological features of a nanoparticle. Spherical aggregates were found by TEM for nanoparticles prepared with the longest PSS investigated at a 0.4 [n+/n−] ratio (Fig. 3). The size of these PIC nanoparticles when analysed by TEM (267 ± 72 nm, *n* = 90) was bigger than their *D*_*H*_ in solution (119 nm ± 28%), suggesting that they could be collapsing upon drying during sample preparation for TEM analysis^40^. TEM imaging of nanoparticles prepared at the same ratio for the shortest PSS evaluated did not reveal any clear morphology (Fig. S1†), suggesting that these nanoparticles were not stable during the TEM sample preparation.

**Figure 4:**
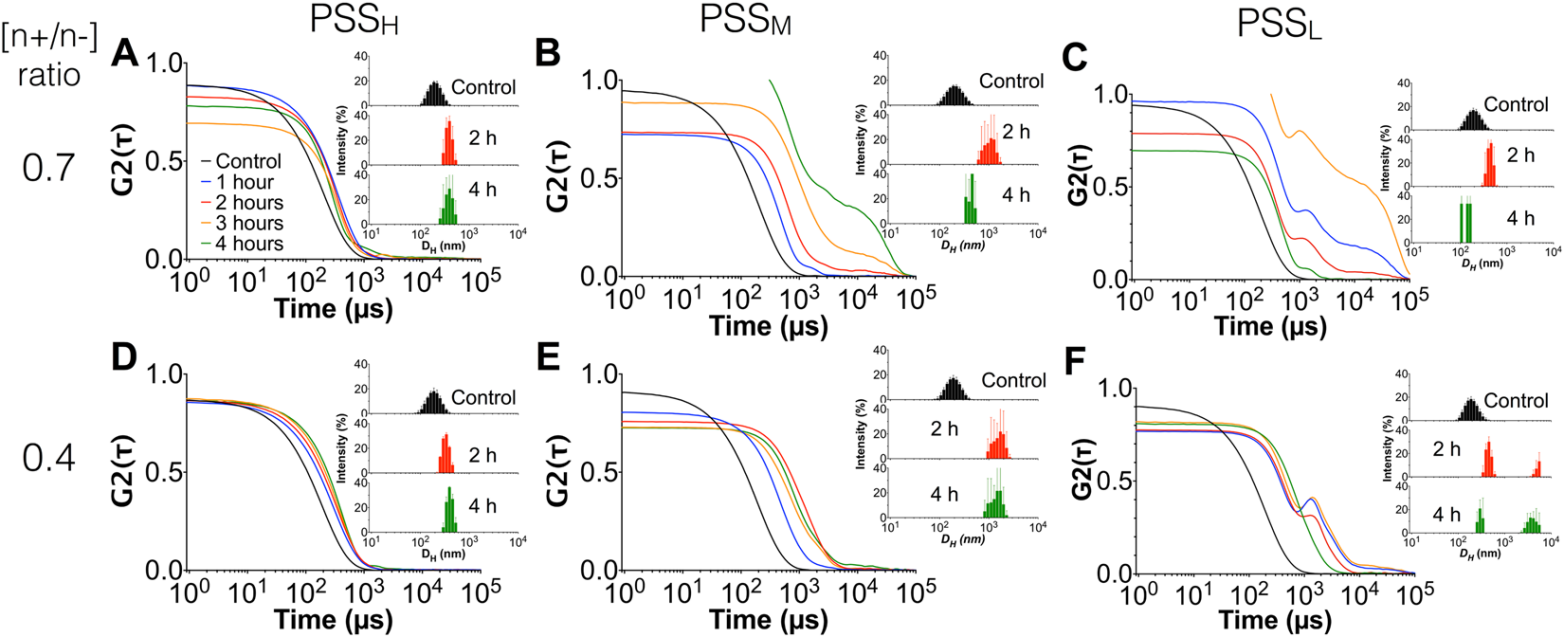
DLS autocorrelation function (ACF) curves and representative size-intensity plots (inset) of PIC nanoparticles prepared from PSS_H_ (**A**,**D**), PSS_M_ (**B**,**E**) and PSS_L_ (**C**,**F**) at [n+/n−] ratios of 0.7 (**A**–**C**) and 0.4 (**D**–**E**) in the absence (control) and presence of 154 mM NaCl (1–4 hours).

We then focused on assessing the stability of the prepared PIC nanoparticles in physiological conditions. Since small electrolytes in solution can shield the ionic networks that cross-link PIC nanoparticles, it is expected that the salts present in biological fluids can compromise the integrity of these nanoparticles. In order to test their stability, all the formulations described above were incubated in 154 mM NaCl at 37 °C and pH 7.4 to simulate physiological conditions, and DLS was monitored over time. For clarity purposes, only the DLS data for PIC nanoparticles prepared at 0.7 and 0.4 [n+/n−] ratios will be discussed next, representing high and low Pol-B loading respectively. The DLS data for all the other nanoparticle formulations evaluated can be found in Fig. S2–S4†, and overall showed very similar profiles to either 0.7 or 0.4 [n+/n−] ratios, depending on the value to which they were closer. Also, formulations at [n+/n−] ratios of 0.1 showed extremely poor stability to salt as expected from their low Pol-B loading and hence weak electrostatic cross-linking of the complex. All PIC nanoparticles swelled in the presence of 154 mM NaCl, due to an increase in electrostatic repulsion as small electrolytes shield the electrostatic attraction between the oppositely charged components of these nanomaterials. This process can be assisted by the association of nanoparticles as they become colloidally unstable, resulting in an overall increase in size^41, 42^. For the reported nanoparticles in this manuscript, this behaviour was strongly influenced by the DP of the polymer. Formulations containing the longest polyelectrolyte, PSS_H_, showed almost no changes in their autocorrelation functions (ACFs) and size distributions by DLS, with only a 2-fold increase in size after 4 hours (Fig. 4). However, PSS_M_ and PSS_L_ experienced more pronounced swelling, increasing 7 and 20 times their initial hydrodynamic diameters after 4 hours, respectively. Whereas PSS_H_ did not show any difference between [n+/n−] ratios, probably due to the strong complexation of Pol-B by this polymer, PSS_M_ and PSS_L_ formed more stable complexes at a 0.4 [n+/n−] ratio than at 0.7. This lack of stability was evident from the loss of the characteristic sigmoidal profile in the ACF curves of these nanoparticles (e.g. Fig. 4B *vs* E), which can be attributed to these nanoparticles breaking down into amorphous debris, as suggested by the smaller-size population found in their intensity plots after 4 hours (Fig. 4B,C).

**Figure 5:**
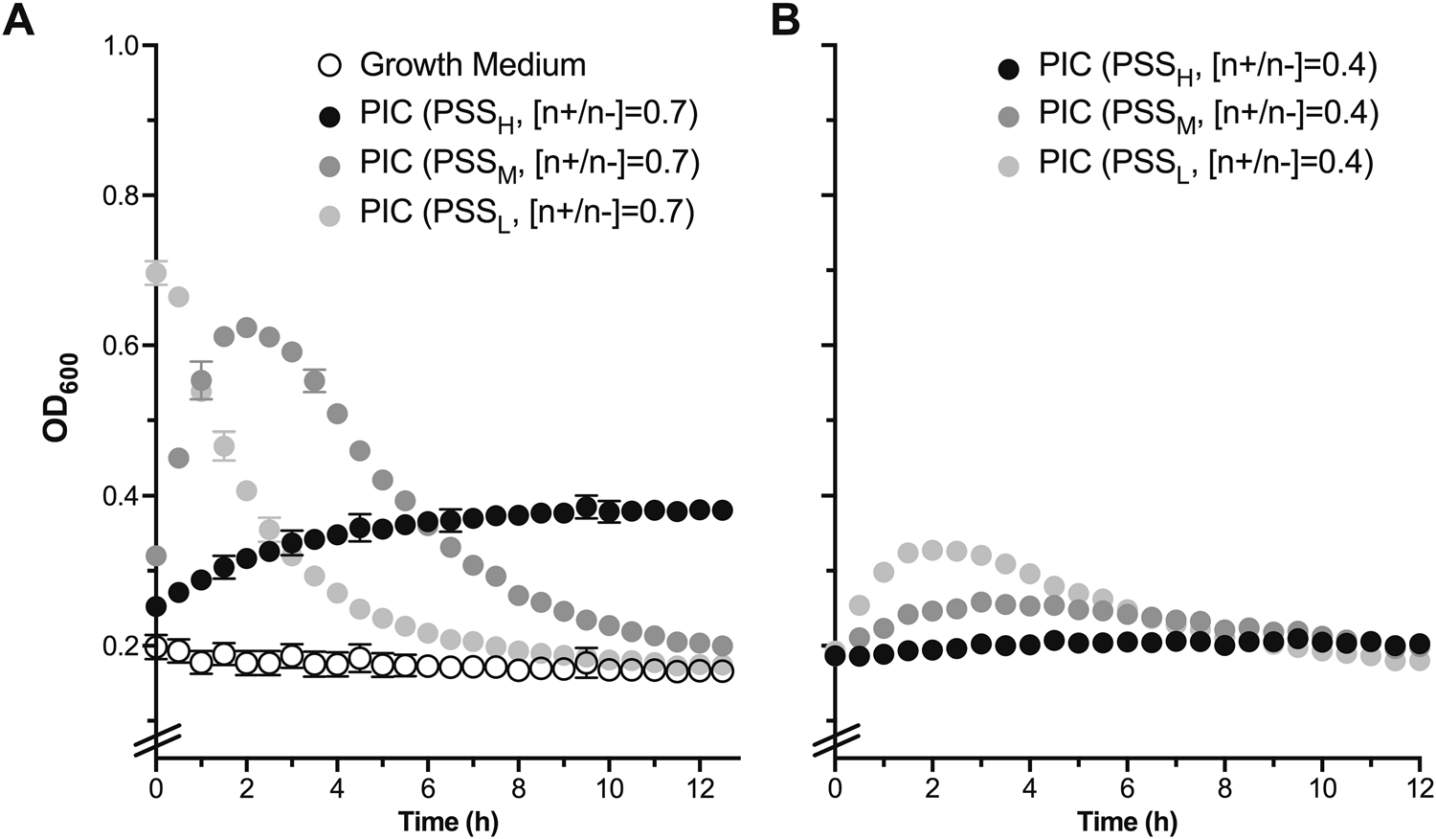
Changes in OD_600_ for 1:1 LB:HEPES growth medium in the absence (**A**, ○) and presence of PIC nanoparticles prepared at a 0.7 (**A**) and 0.4 (**B**) [n+/n−] ratio. Error bars represent the standard deviation, *n* = 3.

Having characterised the stability under simulated physiological conditions of the different formulations prepared, we then evaluated the swelling and stability of these particles in the growth medium used for the antimicrobial assays. This behaviour can be inferred by measuring the changes to the optical density at 600 nm (OD_600_) of particle suspensions in growth medium, as demonstrated in our previous work^15^. Experiments were focused on particles prepared at 0.7 and 0.4 [n+/n−] ratios, because the stability under physiological conditions demonstrated that these formulations were representative examples of the whole series. All nanoparticle formulations increased the OD_600_ when incubated in biological medium, and this effect was dependent on the Pol-B content and the degree of polymerisation of PSS (Fig. 5). Overall, formulations prepared at a 0.7 [n+/n−] ratio increased the turbidity of the medium much more dramatically than their analogues at 0.4, while decreasing the size of the polyelectrolyte resulted in formulations that increased the optical density of the medium faster and to a greater extent (e.g. OD_600_ up to 0.7 in the presence of nanoparticles prepared with PSS_L_ at a 0.7 [n+/n−] ratio). For instance, nanoparticles prepared with PSS_H_ at a 0.7 [n+/n−] ratio showed a steady increase in OD_600_, suggesting that these nanoparticles were swelling or aggregating, but remained stable under assay conditions. On the other hand, particles prepared with the smaller polyelectrolytes, PSS_M_ and PSS_L_, showed a bigger increase in OD_600_, with nanoparticles prepared with PSS_L_ giving an initial OD_600_ that was more than 3.5x bigger than that of the growth medium (Fig. 5). However, suspensions prepared with these smaller polyelectrolytes quickly lost their turbidity, suggesting that the nanoparticles were eventually breaking apart under these conditions and releasing the antimicrobial. The rate of degradation was also dependent on the DP of the PSS, with nanoparticles prepared at a 0.7 [n+/n−] ratio with PSS_L_ recovering optical densities similar to that of the medium after 9 h, while up to 12 h were required for nanoparticles prepared under the same conditions with PSS_M_ (Fig. 5, left). This data was in agreement with the stability observed under simulated physiological conditions (Fig. 4), with formulations prepared with PSS_H_ giving more stable nanoparticles that swelled less.

**Figure 6:**
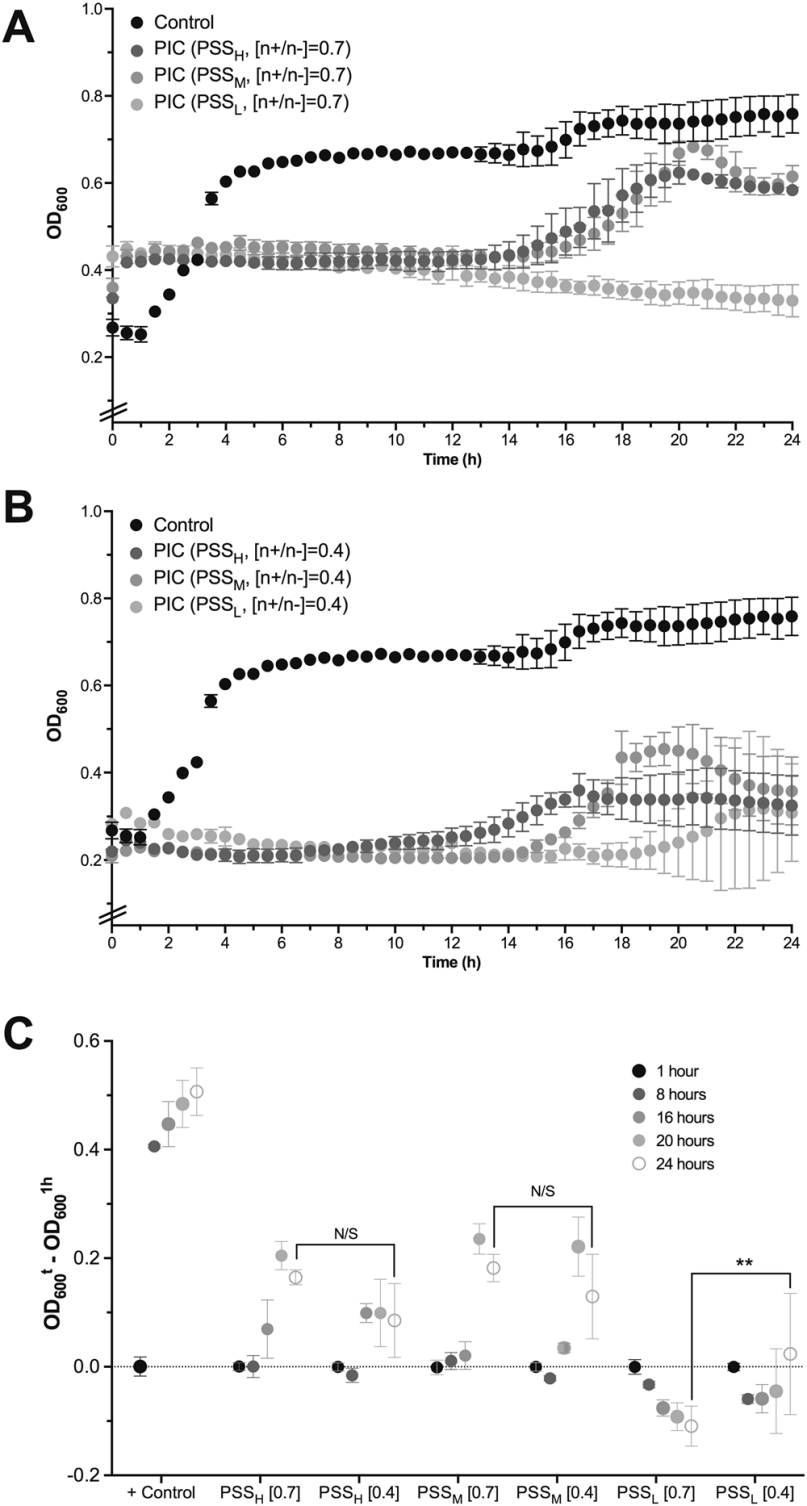
Change in optical density at 600 nm (OD_600_) for *P*. *aeruginosa* cultures in the absence (⦁) and presence of PSS containing PIC nanoparticles prepared at 0.7 (**A**) or 0.4 (**B**) [n+/n−] ratios. Error bars represent the standard deviation, *n* = 3. (**C**) Representative relative changes in OD_600_ from *P*. *aeruginosa* cultures in the absence (+Control) and presence of PSS containing PIC nanoparticles prepared at 0.7 or 0.4 [n+/n−] ratios, indicated in brackets. **p < 0.01, ‘N/S’ = not significant (two-way ANOVA, Tukey test, CI = 95%). Error bars represent the standard deviation, *n* = 3.

We then investigated the effect of PIC nanoparticles and their individual components on *P*. *aeruginosa* cultures by monitoring as well their OD_600_ over time. This is a routine technique used in microbiology that correlates any increase in the turbidity of a sample to bacterial growth^43^. The growth of *P*. *aeruginosa* was not inhibited by the presence of the polymers (Fig. S6†, PSS_L_ and PSS_H_ shown as representative examples), but incubation with Pol-B at the concentrations found in PIC nanoparticles at 0.7 and 0.4 [n+/n−] ratios (87.5 and 50 µM, respectively) completely inhibited the growth of this bacterium for the duration of this experiment (Fig. S6†). To facilitate the visual comparison of these antimicrobial results, the OD_600_ values of *P*. *aeruginosa* cultures in the presence of PIC nanoparticles were normalised against the optical density observed one hour after the start of the experiment, when the initial increase in OD_600_ attributed to PIC nanoparticle swelling had stabilised (Fig. 6A,B), and plotted as a function of the polymer and formulation used at representative time points (Fig. 6C).

Decreasing the DP of PSS had a dramatic effect on the antimicrobial activity of the nanoparticles. Our previously reported particles could inhibit the growth of *P*. *aeruginosa* up to 14.5 h^15^, and similar effects were observed with the nanoparticles prepared with PSS_H_ and PSS_M_ at a 0.7 [n+/n−] ratio (Fig. 6A). However, PIC nanoparticles prepared with the shortest polymer, PSS_L_, at the same [n+/n−] ratio showed a remarkable antimicrobial effect, with the turbidity at all times below that observed at the beginning of the experiment and no evidence of *P*. *aeruginosa* growth for the duration of the experiment (Fig. 6A,C). We believe this is a consequence of the lower stability of this formulation, that is able to maintain therapeutic levels of Pol-B throughout the duration of the experiment. Attempts to characterise the rate of release of Pol-B from these nanoparticles were compromised by the slow diffusion of this antimicrobial across the dialysis devices evaluated (Fig. S5), something we had observed for our previously reported nanoparticles^15^. Reducing the amount of antimicrobial had a detrimental effect on the antimicrobial activity of the nanoparticles, with formulations prepared with PSS_L_ at a 0.4 [n+/n−] ratio showing signs of microbial growth after 20 hours of incubation (Fig. 6B and C). Nanoparticles prepared with PSS_M_ and PSS_H_ at this [n+/n−] ratio displayed an even weaker antimicrobial effect and after 14 hours the growth of the pathogen was evident again. Overall, nanoparticles containing PSS_M_ and PSS_H_ showed very similar antimicrobial activities regardless of their [n+/n−] ratio, and in general displayed no significant differences in the turbidity of bacterial cultures over time. These results are in agreement with the distinct physiological stability observed for these nanomaterials (Fig. 4), with PSS_L_ at a 0.7 [n+/n−] ratio forming the least stable nanoparticles with the strongest antimicrobial activities across the range of DPs and [n+/n−] ratios tested.

**Figure 7:**
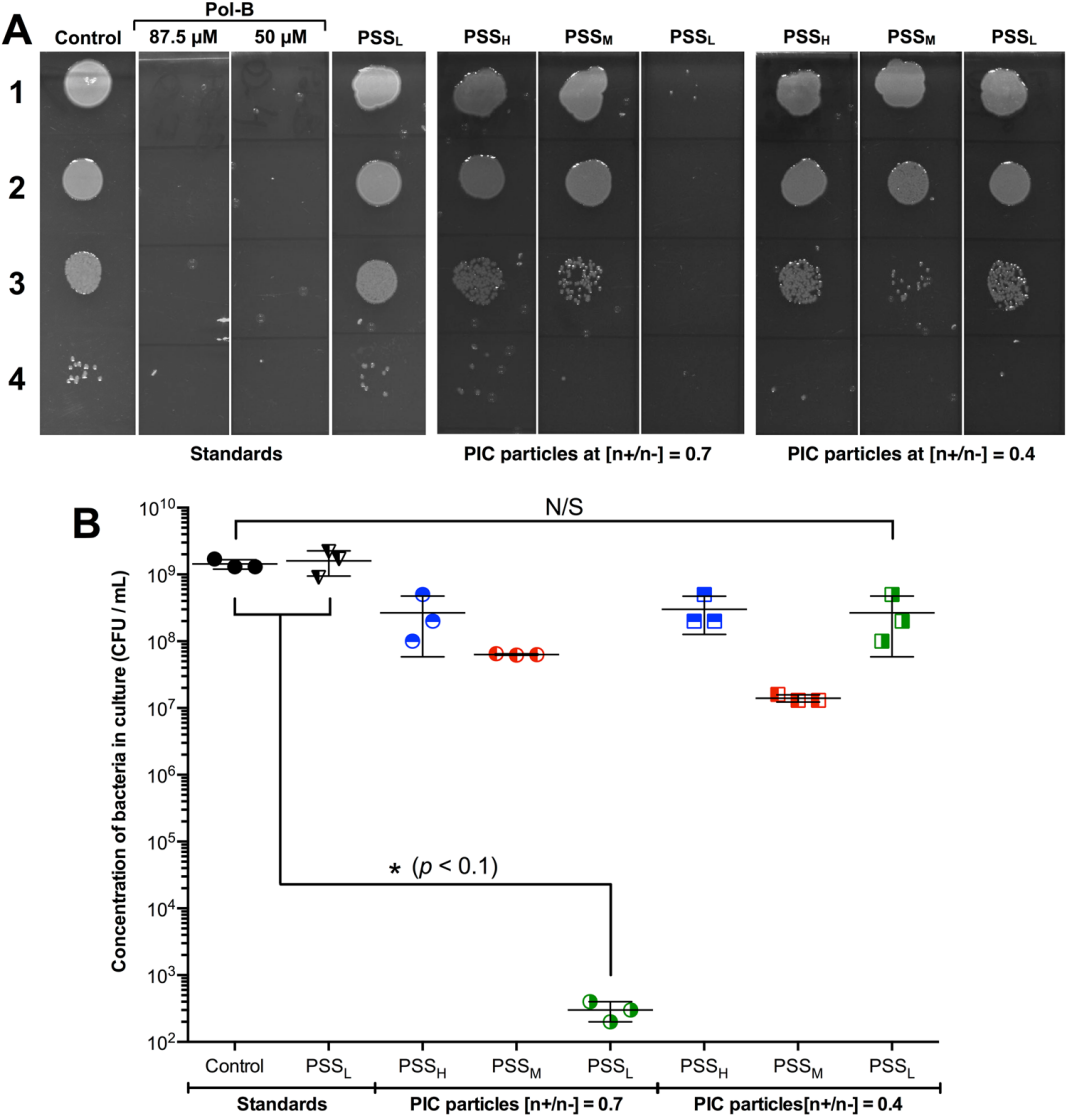
(**A**) Representative agar plates used to evaluate antimicrobial activity of PIC nanoparticles prepared from PSS and Pol-B. Lane 1: No dilution, lane 2: 10^2^-fold dilution, lane 3: 10^4^-fold dilution, and lane 4: 10^6^-fold dilution. (**B**) CFU/mL of *P*. *aeruginosa* in the absence (control) and presence of PIC nanoparticles prepared from different PSS sources at different [n+/n−] ratios, calculated from the colonies found in the agar plates (one-way ANOVA, Tukey test, CI = 95%). ‘N/S’ = not significant. Error bars represent the standard deviation, *n* = 3.

Samples from these growth assays were collected after 24 hours of incubation and spotted on agar plates to quantify the number of viable bacteria remaining at this stage^43^. Several dilutions were spotted on agar plates to identify suitable conditions for colony counting. *P*. *aeruginosa* was able to grow under these conditions and we observed that PSS_L_ (as a representative polymer) had no effect on this growth (Fig. 7A). Both concentrations of Pol-B used to prepare the nanoparticles completely eradicated *P*. *aeruginosa* from the culture (control). Interestingly, all formulations displayed some degree of bactericidal effect, as evidenced by the decrease of approximately two orders of magnitude in the number of viable bacterial colonies found for the least potent formulations. These results were similar to those observed before for the 70 kDa PSS^15^, that showed also a decrease of two orders of magnitude in microbial viability, and in agreement with turbidity experiments (Fig. 6). Nanoparticles prepared at a 0.7 [n+/n−] ratio with the shortest polyelectrolyte, PSS_L_, were again those that showed the biggest antimicrobial effect. Remarkably, the number of viable colonies for this formulation was reduced more than six orders of magnitude (Fig. 7B), suggesting that these nanoparticles were over 10,000 times more efficient in killing *P*. *aeruginosa* than any of the other ones we have reported.

## Conclusions

Here, we report the preparation and characterisation of Pol-B containing PIC nanoparticles using three different PSS selected across a wide range of degrees of polymerisation. We have assessed the physiological stability of these nanoparticles and their antimicrobial activity against *P*. *aeruginosa*, an opportunistic pathogen currently treated with Pol-B. Our results indicate that the antimicrobial activity of these nanoparticles can be significantly increased by tuning the degree of polymerisation of the polyelectrolyte component, in this case PSS. Nanoparticles prepared with the PSS with the smallest DP showed the highest antimicrobial activity. The antimicrobial activity of the nanoparticles prepared with PSS of this smallest degree of polymerisation was dependent of the [n+/n−] ratio, an effect not found in formulations prepared from longer polymers. Overall, tailoring the degree of polymerisation o﻿f PSS and the [n+/n−] ratio, we have been able to identify a PIC nanoparticle formulation that shows a sustained inhibitory effect in the growth of *P*. *aeruginosa* and can reduce the number of viable colonies of this pathogen significantly more effectively than our previous reported formulation. We believe our results highlight the importance of selecting a correct polyelectrolyte and formulation to tune the stability and antimicrobial activity, and should be of value in the future development of these materials as a platform for the delivery of last-resort antimicrobials. Our efforts to further develop these nanomaterials, and tailor composition, formulation and stability for *in vivo* and *ex vivo* applications will be reported in due course.

## Electronic supplementary material

Supplementary Information 
