# Supplementary material for: Polymyxin B containing polyion complex (PIC) nanoparticles: Improving the antimicrobial activity by tailoring the degree of polymerisation of the inert component
Source: Sci Rep. 2017 Aug 24;7:9396. doi: 10.1038/s41598-017-09667-3 (PMC5570901; doi:10.1038/s41598-017-09667-3)
Supplement: Supplementary file 1 — Supplementary Information [file 41598_2017_9667_MOESM1_ESM.pdf]

Polymyxin B containing polyion complex (PIC) nanoparticles: Improving the antimicrobial activity by tailoring the degree of polymerisation of the inert component

Ignacio Insua,[a,c] Laimdota Zizmare, [a,c] Anna F. A. Peacock,[a] Anne Marie Krachler,[b,c,§] and Francisco Fernandez-Trillo*[a,]

[a]School of Chemistry, [b]School of Biosciences and [c]Institute of Microbiology and Infection, University of Birmingham, B15 2TT Birmingham, UK

§ Current address: Department of Microbiology and Molecular Genetics, University of Texas McGovern Medical School at Houston, Houston, TX, 77030, USA.

TABLE OF CONTENTS

1. CHARACTERISATION OF PIC NANOPARTICLES 2

1.1. SIZE AND CHARGE 2
1.2. ADDITIONAL TEM MICROSCOPY 3

2. STABILITY OF PIC NANOPARTICLES UNDER SIMULATED PHYSIOLOGICAL CONDITIONS 4

3. DIFFUSION OF POL-B ACROSS DIALYSIS MEMBRANES 7

4. ANTIMICROBIAL ACTIVITY OF PSS AND POL-B 8

4.1. CONTROL GROWTH CURVES 8

1. CHARACTERISATION OF PIC NANOPARTICLES

1.1. Size and charge

Table S1. Hydrodynamic diameter (D_H) and ζ-potential of PIC nanoparticles prepared from six different polymers in combination with Pol-B at ten different [n+/n-] ratios.

Polymer	[n+/n-] ratio	D_H ± SD (nm)	PdI [b]	ζ-potential ± SD (mV) [a]	Notes
PSS_H	1.0	-	-	-	Flocculation
	0.9	-	-	-	Flocculation
	0.8	-	-	-	Flocculation
	0.7	199 ± 58	0.08	-55.1 ± 10.7	-
	0.6	194 ± 58	0.09	-54.7 ± 9.9	-
	0.5	196 ± 61	0.10	-56.8 ± 10.4	-
	0.4	193 ± 67	0.12	-51.4 ± 12.4	-
	0.3	190 ± 60	0.10	-52.7 ± 9.2	-
	0.2	193 ± 71	0.14	-50.3 ± 11.0	-
	0.1	182 ± 58	0.10	-46.6 ± 14.2	-
PSS_M	1.0	-	-	-	Flocculation
	0.9	-	-	-	Flocculation
	0.8	201 ± 57	0.08	-49.5 ± 7.3	-
	0.7	202 ± 61	0.09	-48.5 ± 8.5	-
	0.6	197 ± 59	0.09	-50.0 ± 8.8	-
	0.5	202 ± 73	0.13	-49.8 ± 7.9	-
	0.4	197 ± 62	0.10	-49.5 ± 9.1	-
	0.3	192 ± 55	0.08	-49.6 ± 9.3	-
	0.2	179 ± 62	0.12	-48.1 ± 9.7	-
	0.1	180 ± 65	0.13	-49.2 ± 9.6	-
PSS_L	1.0	-	-	-	Flocculation
	0.9	-	-	-	Flocculation
	0.8	196 ± 60	0.09	-45.7 ± 9.3	-
	0.7	201 ± 72	0.13	-46.5 ± 8.1	-
	0.6	189 ± 49	0.07	-46.7 ± 7.1	-
	0.5	194 ± 68	0.12	-48.9 ± 8.9	-
	0.4	193 ± 60	0.10	-49.5 ± 9.2	-
	0.3	186 ± 56	0.09	-46.8 ± 8.0	-
	0.2	185 ± 56	0.09	-48.0 ± 9.2	-
	0.1	182 ± 53	0.08	-47.9 ± 9.4	-

[a] SD indicates the standard deviation found for the only size or charge population fitted by the software.
[b] Polydispersity Index (PdI) calculated using the formula: PdI = $(SD/D_H)^2$.

1.2. Additional TEM microscopy

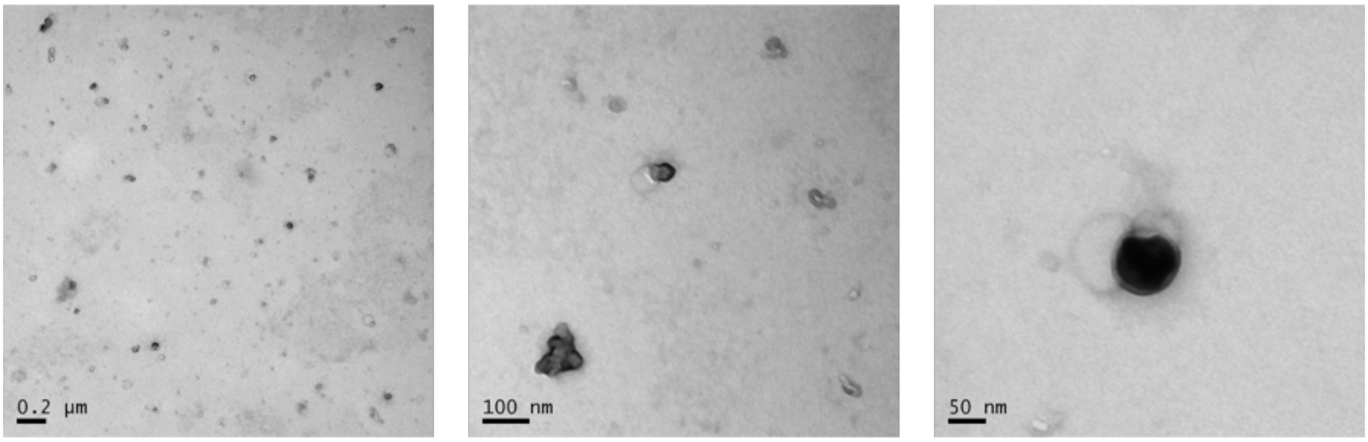

Fig. S1 TEM micrographs of PIC nanoparticles prepared from Pol-B and PSS_L at a 0.4 [n+/n-] ratio.

2. STABILITY OF PIC NANOPARTICLES UNDER SIMULATED PHYSIOLOGICAL CONDITIONS

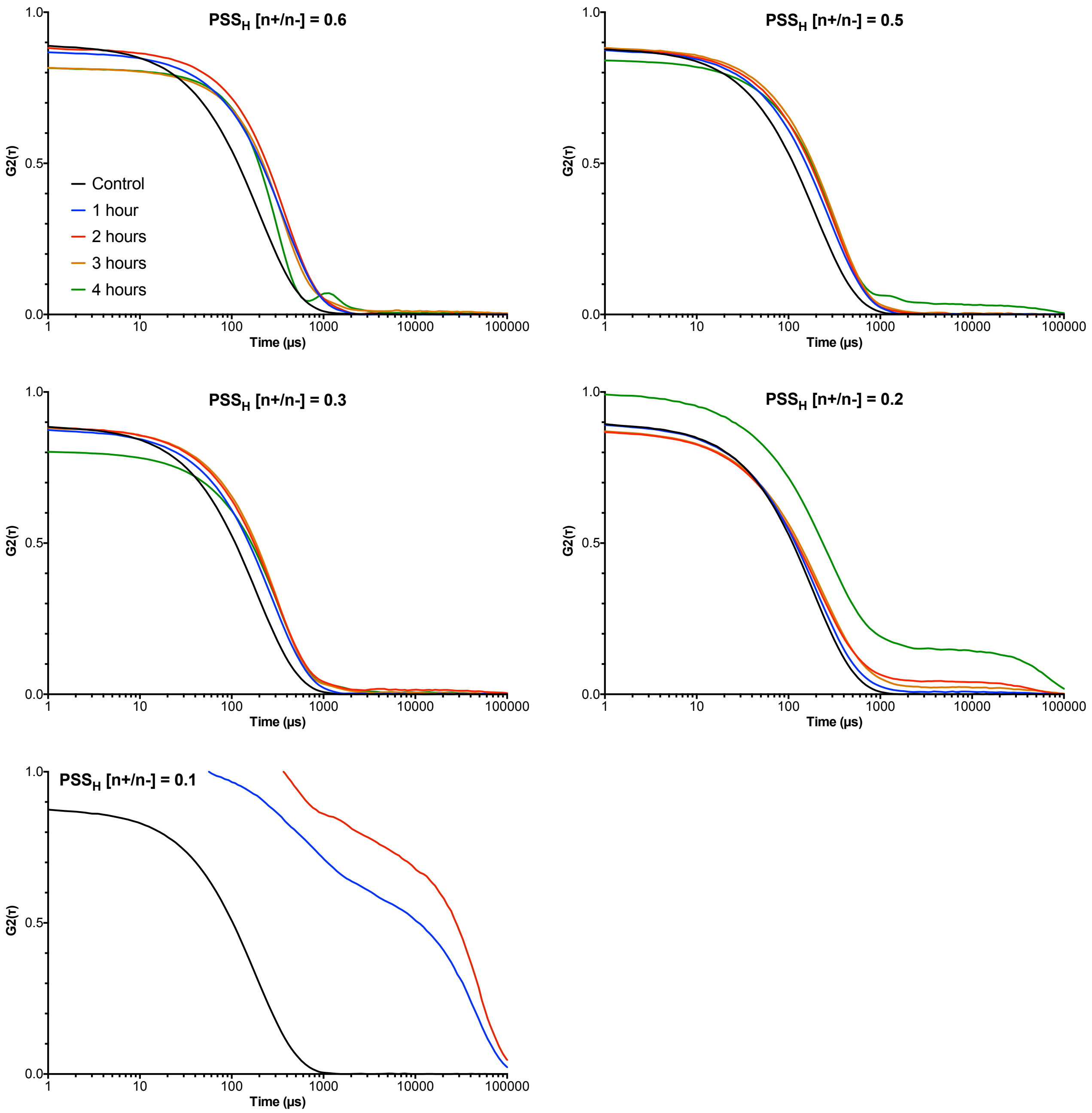

Fig. S2 DLS autocorrelation function (ACF) curves of PIC nanoparticles prepared from PSS_H at different [n+/n-] ratios in the absence (control) and presence of 154 mM NaCl over time (1-4 hours).

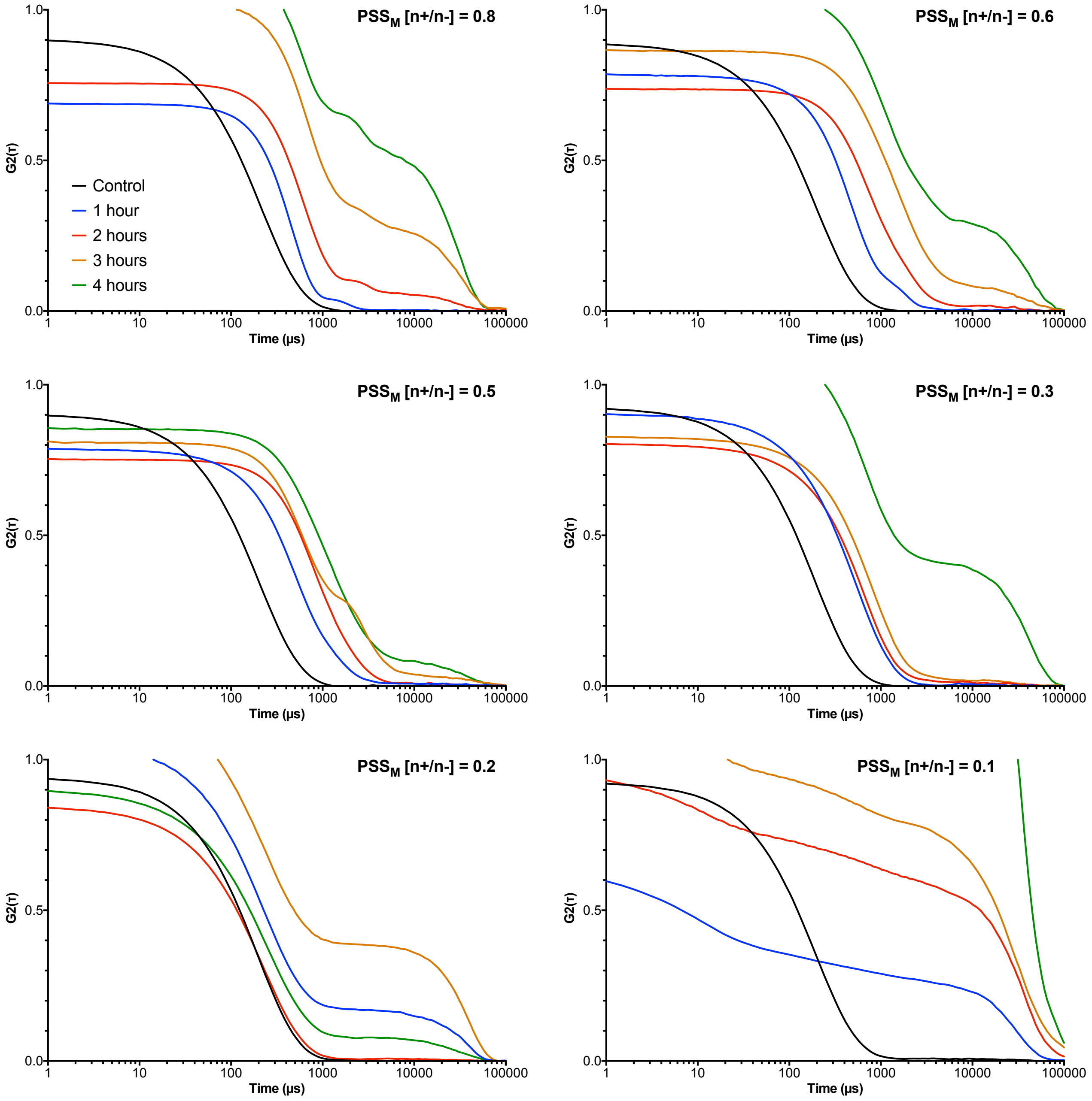

Fig. S3 DLS autocorrelation function (ACF) curves of PIC nanoparticles prepared from PSS$_M$ at different [n+/n-] ratios in the absence (control) and presence of 154 mM NaCl over time (1-4 hours).

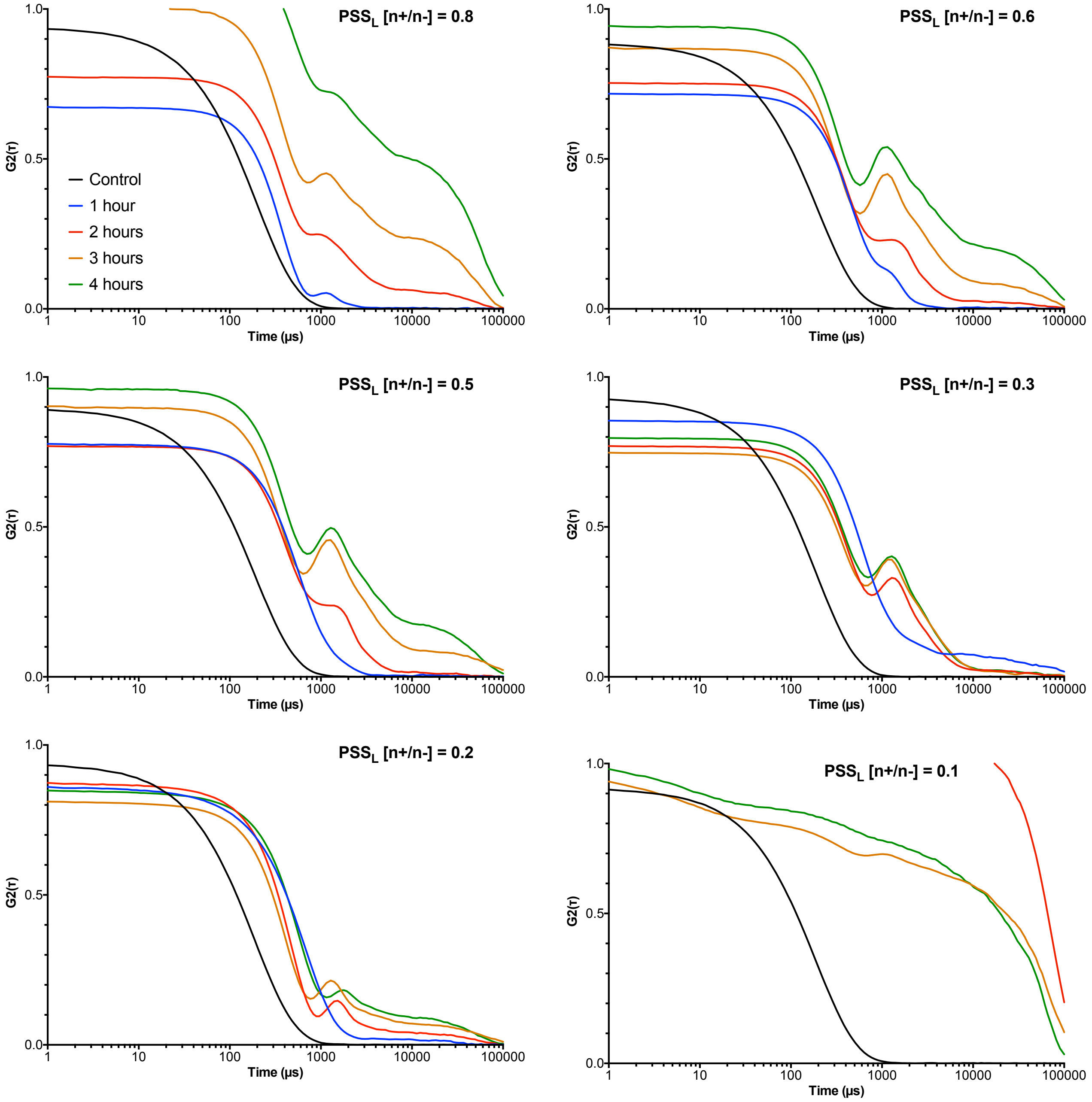

Fig. S4 DLS autocorrelation function (ACF) curves of PIC nanoparticles prepared from PSS$_L$ at different [n+/n-] ratios in the absence (control) and presence of 154 mM NaCl over time (1-4 hours).

3. DIFFUSION OF POL-B ACROSS DIALYSIS MEMBRANES

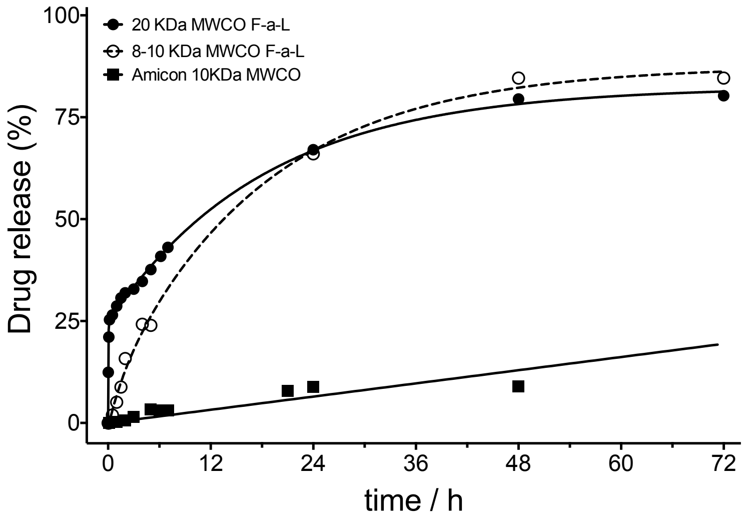

Fig. S5 Pol-B content found in the dialysate of 175 µM solutions of Pol-B in 5 mM HEPES buffer at pH 7.4. Content was normalised to that found in a 175 µM Pol-B sample (drug loading in these PIC nanoparticles) diluted down to the total dialysis volume (100%). *n* = 3.

4. ANTIMICROBIAL ACTIVITY OF PSS AND POL-B

4.1. Control growth curves

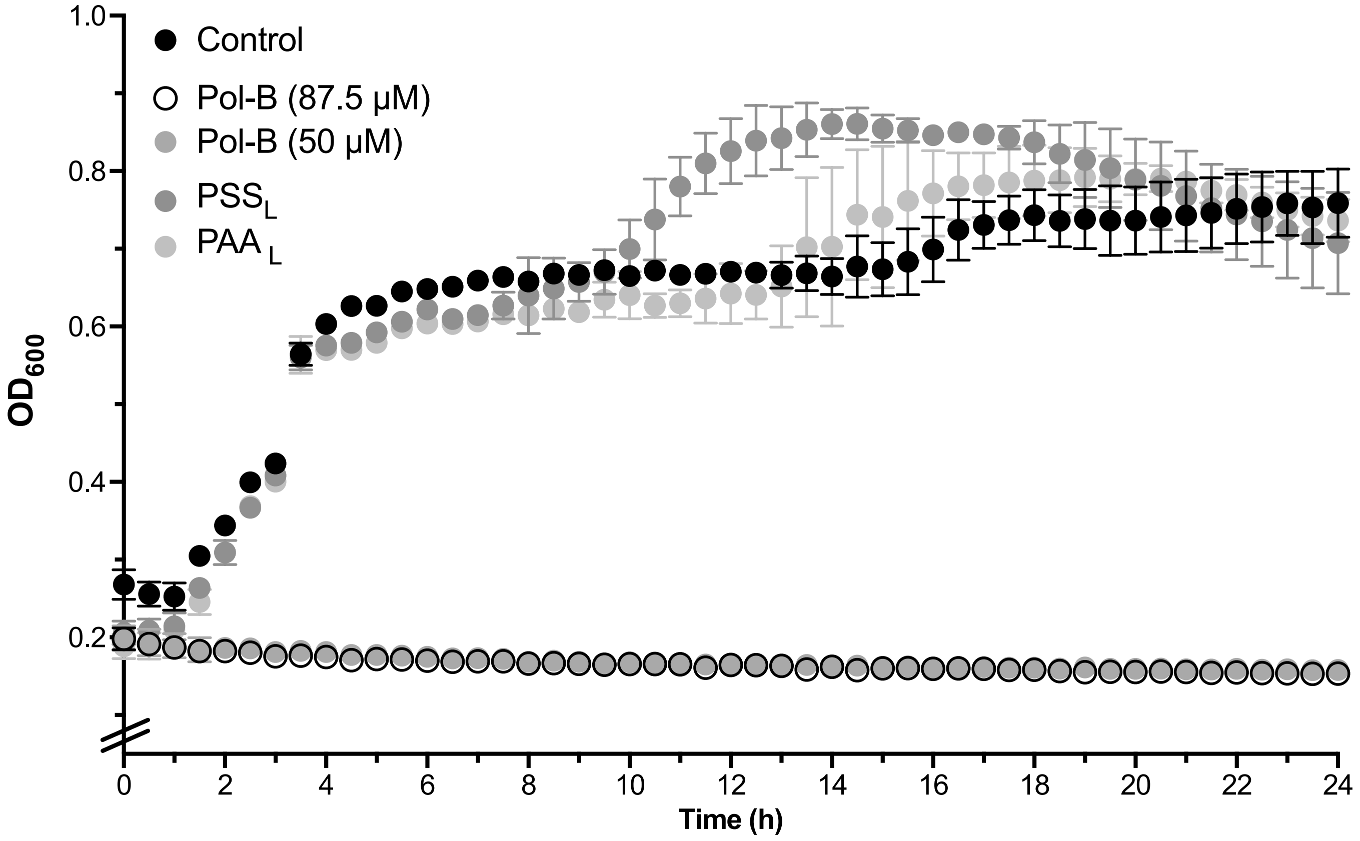

Fig. S6 Change in optical density at 600 nm (OD_{600}) for *P. aeruginosa* cultures in the absence (●) and presence of Pol-B, PSS_L, PSS_H. Error bars represent the standard deviation, *n* = 3.
